# Case Report: Crossing a rugged road in a primary immune regulatory disorder

**DOI:** 10.3389/fped.2022.1055091

**Published:** 2023-01-09

**Authors:** Mayla Sgrulletti, Cristina Cifaldi, Silvia Di Cesare, Barbara Kroegler, Elisabetta Del Duca, Valentina Ferradini, Simona Graziani, Mario Bengala, Gigliola Di Matteo, Viviana Moschese

**Affiliations:** ^1^ Pediatric Immunopathology and Allergology Unit, Policlinico Tor Vergata, University of Tor Vergata, Rome, Italy; ^2^ PhD Program in Immunology, Molecular Medicine and Applied Biotechnology, University of Rome Tor Vergata, Rome, Italy; ^3^ Academic Department of Pediatrics, Immune and Infectious Diseases Division, Research Unit of Primary Immunodeficiencies, Bambino Gesù Children's Hospital, IRCCS, Rome, Italy; ^4^ Department of Systems Medicine, University of Tor Vergata, Rome, Italy; ^5^ Rheumatology Allergology and Clinical Immunology, Department “Medicina dei Sistemi”, University of Rome Tor Vergata, Rome, Italy; ^6^ Department of Biomedicine and Prevention, University of Rome Tor Vergata, Rome, Italy; ^7^ Laboratory of Medical Genetics, Tor Vergata Hospital, Rome, Italy

**Keywords:** inborn errors of immunity, primary immunodeficiency, secondary hypogammaglobulinemia, rheumatic disease, immune dysregulation

## Abstract

Over the last decades, Inborn Errors of Immunity (IEI) characterized by an immune dysregulatory picture, isolated or combined with infections, have been increasingly identified and referred as Primary Immune Regulatory Disorders (PIRD). PIRD diagnosis may be difficult due to heterogeneity of time onset, sequence of clinical manifestations and laboratory abnormalities. Moreover, the dissection of a PIRD vs. a secondary immunodeficiency (SID) might be a real challenge since the same indications for immunosuppressant treatments might represent *per se* a PIRD clinical expression. Here we report a female patient with a history of recurrent respiratory and urinary tract infections since early infancy and a diagnosis of Rheumatoid Arthritis in adulthood. After poor response to several biologicals she was treated with Rituximab and sent to immunology referral for a severe hypogammaglobulinemia. Clinical and immunological features matched a diagnosis of common variable immunodeficiency and when IgG replacement therapy and antibiotic prophylaxis were added a good infectious control was obtained. Next generation sequencing analysis has revealed a novel heterozygous VUS in the *IKBKB* gene (c.1465A > G; *p*.Ser489Gly). Functional analysis has shown a reduced capacity of B lymphocytes and CD4 positive T cells in inducing I*κ*B*α* degradation, with negative impact on NF-kB pathway. Due to recurrent infections attributed to a common condition in childhood and to an exclusive autoimmunity-centered approach in adulthood, both diagnosis and suitable treatment strategies have suffered a significant delay. To reduce the diagnostic delay, pediatricians, general practitioners and specialists should be aware of IEI and the challenges to differentiate them from SID. Furthermore, genetic characterization and functional analysis may contribute to a personalized approach, in a perspective of targeted or semi-targeted therapy.

## Introduction

Primary Immunodeficiencies, most recently termed as “Inborn errors of immunity” (IEI), refer to a wide group of inherited defects of one or more component of the innate and/or adaptive immune system (1). Susceptibility to recurrent and/or severe infectious diseases that take longer to resolve or need for hospitalization, use of intravenous antibiotics, atypical infections for localization or causing pathogen, historically represent warning signs for IEI (2). On the other hand, infections are common in childhood, so that accurate family and personal history together with anatomical and environmental factors should be considered before the suspicion of an IEI. Over the last decade, it has been increasingly acknowledged that immune-dysregulation, expressed with autoimmunity, hyper/auto-inflammation, granulomas, lymphoproliferative disorders and malignancies, might occur in patients with IEI (3). The term Primary Immune Regulatory Disorders (PIRD) coins a subset of IEI with non-infectious immune-mediated pathology stemming from cellular and molecular mechanisms leading to immune tolerance failure (4, 5). The recognition of molecular mechanisms underlying PIRDs is paving the way for targeted or semi-targeted therapeutic approaches (3). Approximately 20% of IEI patients might suffer from isolated immune dysregulation or combined with infectious recurrence (6). Diagnosis of a PIRD may be cumbersome due to their heterogeneity in terms of time onset, sequence of clinical manifestations and laboratory abnormalities. Not every infectious history or dysregulation is promptly associated with an IEI causing a significant diagnostic delay and a poor outcome. To make the history even more puzzling, some immunological treatments used for the same clinical indications that might be expressed in a IEI/PIRD condition may cause a iatrogenic secondary immunodeficiency (SID) (7). In fact, iatrogenic SIDs have been exponentially reported (8), in tandem with the progressive increase in the incidence of autoimmune diseases and the wider use of standard immunosuppressant drugs (i.e., corticosteroids and DMARDs) as well as biological therapies (bDMARDs) in several specialty settings (i.e., hematological, rheumatological, neurological, etc). These conditions mainly derive from B-cell targeting biologicals, with the anti-CD20 monoclonal antibody rituximab (RTX) as the cornerstone (9). Delayed manifestations of genetic immunological disorders and the increasing recognition of patients with previously undiagnosed IEI receiving biologics, renders the dissection of a IEI vs. a SID extremely challenging. Increasing awareness among the different subspecialties and appropriate investigation will favor early diagnosis as well as optimal treatment for a better outcome and quality of life.

Here we report the case of a currently 47-year-old female patient who suffered with recurrent and severe infections since early infancy and developed in adulthood a Rheumatoid Arthritis with poor response to several biological DMARDs. Immunology referral allowed a clinical diagnosis of Common Variable Immunodeficiency (CVID) and targeted Next Generation Sequencing (NGS) analysis identified a novel heterozygous variant in *IKBKB* gene. This study aims to reinforce the notion that pediatricians, general pratictioners (GPs) and different subspecialties should be aware of the diverse temporality and spectrum of IEI/PIRD disorders and enhance a multidisciplinary approach for continual improvement in the field.

## Materials and methods

The work was conducted in accordance with the ethical standards of the institutional research committee and with the 1964 Helsinki declaration and its later amendments or comparable ethical standards. The patient gave her informed consent to perform immunological and genetic analysis. According to best general practice, immunological work-up included the evaluation of serum Ig level by nephelometry, serum IgG subclasses by radial immunodiffusion, T and B cell immunophenotype, specific IgG antibody response to pneumococcus vaccine, autoantibodies, complement C3 and C4 and specific serum IgE. Targeted Next-Generation sequencing.

DNA was extracted by QIAamp DNA Blood Mini Kit (Qiagen) and prepared, sequenced and analyzed according to manufacturer's protocol as previously reported (10). Our CVID custom Ion Torrent panel, including 62 known genes (Supplementary Table S1), was designed with Ampliseq Designer software using GRCh38 as references. Sanger sequencing on gDNA isolated from total PBMCs was performed to confirm the presence of mutations in *IKBKB* gene (ABI PRISM 3130, Applied Biosystems, Foster City, CA).

### Peripheral blood immunophenotype

All flow cytometric analyses were performed on EDTA blood samples within 24 h of venipuncture. After red blood cell lysis with ammonium chloride, lymphocytes were washed, resuspended in PBS, and stained with the following mouse anti-human antibodies to identify T and B cell subsets: CD45RA (clone T6D11; Miltenyi Biotec), CD3 (clone BW264/56; Miltenyi Biotec), CCR7 (clone 3D12; eBioscience), CD4 (clone OKT4; Becton Dickinson), CD8 PE- (clone RPA-T8; Becton Dickinson), CD19 (clone SJ25C1; Becton Dickinson), CD16 (clone 3G8), CD56, CD27 (clone M-T271, Becton Dickinson), TCR *α*-beta (clone T10B9; Becton Dickinson), TCR gamma-delta (11F3; Miltenyi Biotec), CD21 (clone B-ly4; Becton Dickinson), CD24 (clone ML5; Becton Dickinson), IgD (clone IA6-2; Becton Dickinson), Goat F(ab)2 anti-Human IgM (µ) (Jackson ImmunoResearch), and CD38 (clone HIT2; Becton Dickinson). Cells were incubated with the appropriate antibody cocktail for 30 min at 4 °C and then washed with PBS and resuspended in PBS for flow cytometric acquisition. At least 50,000 events were acquired within the lymphogate. Data were acquired on a FACSCanto II (Becton Dickinson) and analyzed with FlowJo software (Tree Star Inc, version 9.3.2).

### FACS studies

PBMCs from patient and healthy controls were isolated by density gradient centrifugation on Ficoll-Paque PLUS (GE Healthcare), washed twice in PBS, and maintained in complete RPMI (Sigma-Aldrich) containing 10% FBS, 2 mM l-glutamine (Sigma-Aldrich), and 100 U/ml penicillin and streptomycin (Sigma-Aldrich).

p65 phosphorylation: Total PBMC (300.000 per tube) were stimulated with phorbol 12-myristate 13-acetate (PMA; Sigma, Cat# P1585) /Ionomycin (Ionomycin; Sigma Cat #I0634) at 37 °C for 0, 5, 10, 15 min. Stimulation was blocked and cells were fixed using Fix Buffer I (BD Biosciences, Cat #557870,) for 10 min at 37 °C then centrifuged, washed twice with FACS buffer (PBS supplemented with 2% FBS and 1 mM EDTA) and permeabilized for 10 min on ice with 1 ml Phosflow Perm Buffer III (BD Biosciences, Cat #558050). After two washes, the cells were stained for anti-CD4+, anti-CD19 + antibodies and with anti-phospho-p65 (BD Biosciences, Cat #558422) for 30 min at RT. Samples were washed three times and data were collected on FACSCanto II (Becton Dickinson).

I*κ*B*α* degradation: Total PBMC (300.000 per tube) were stimulated with phorbol 12-myristate 13-acetate (PMA)/Ionomycin (PMA 100 ng/ml, Ionomycin 1 *μ*g/ml) at 37 °C for 0, 15, 30, 60 min.

Cells were washed with FACS buffer. Fc receptors (BD Biosciences, Cat#553141) were blocked for 5 min at RT followed by a 20 min of staining on ice with anti- CD4+, CD19+ antibodies and incubated for 30 min at 4 °C. After two washes, cells were permeabilized for 30 min at RT, according to manufacturer's protocol, centrifuged and washed twice with FACS buffer. Cells were stained with anti-monoclonal IкB*α* (H-4) (Santa Cruz Cat #sc-1643) for 30 min at RT then washed and incubated with anti-Mouse IgG (eBiosciences, Cat# 11-4011-85). After two washes, data were collected on FACSCanto II (Becton Dickinson). All Data were analyzed with FlowJo software (Tree Star Inc, version 9.3.2). Data were analyzed with Graph-Pad Prism, version 6.2 (Graph Pad Software, la Jolla, CA).

## Results

### Case presentation

A 47-year-old woman affected with Rheumatoid Arthritis (RA) was referred from the Rheumatology outpatient clinic to the Regional Center for Immunodeficiency Diseases at Tor Vergata University Hospital, Rome, Italy for hypogammaglobulinemia and recurrent infections.

**Figure 1:**
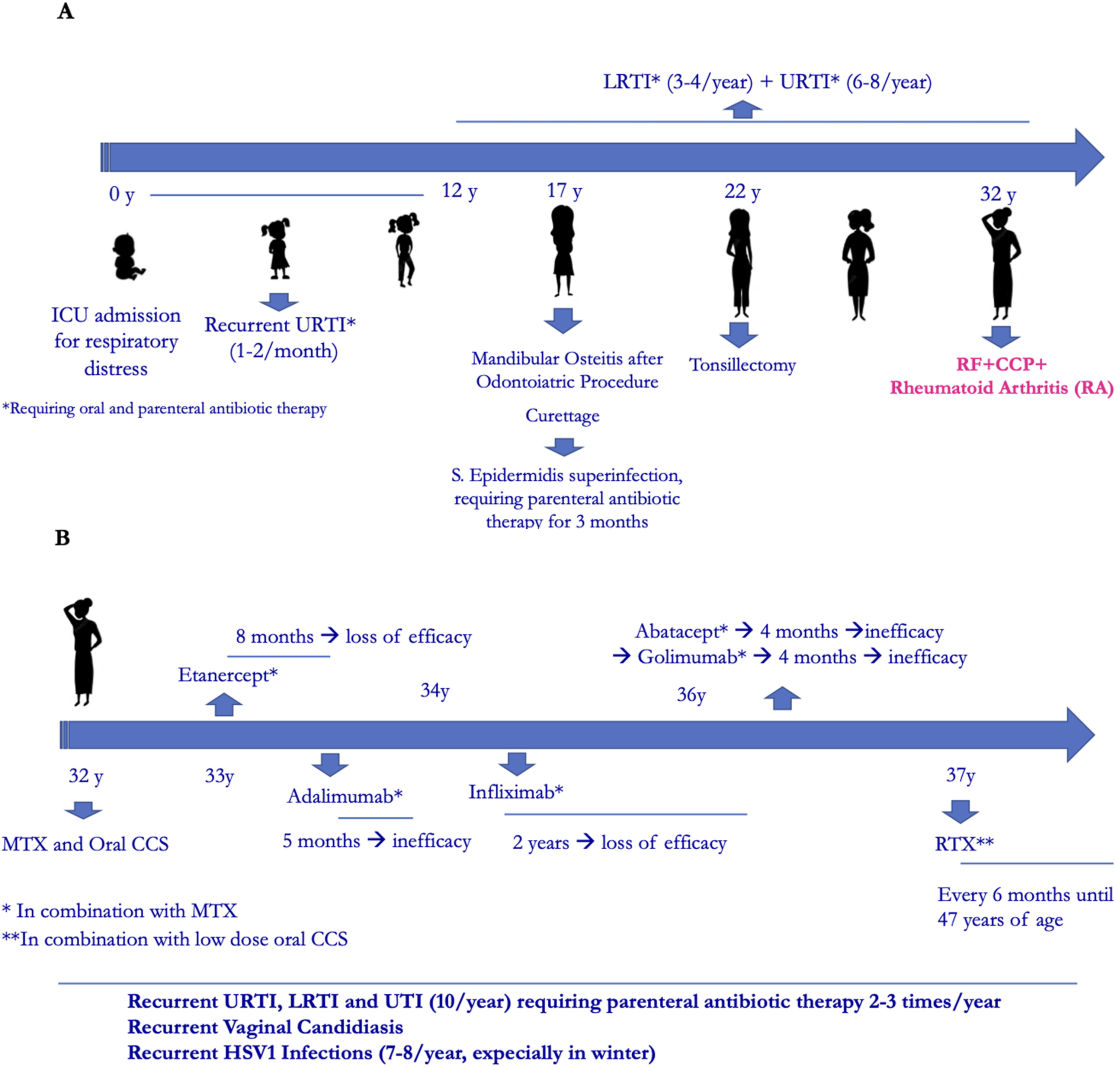
(**A**) Clinical history timeline. (**B**) Rheumatological treatment timeline.

Her family history was positive for Hashimoto thyroiditis, psoriasis, hepatocarcinoma and lymphoma. Past medical history revealed an intensive care unit admission at birth for respiratory distress, with non-invasive ventilation until the first month of life. Since early infancy, she suffered from one or two episodes per month of upper respiratory tract infections (URTI) that repeatedly required oral antibiotic treatment and the use of parenteral antibiotic therapy 2–3 times/year. In adolescenthood, in addition to recurrent URTI, she suffered from recurrent lower respiratory tract infections (LRTI) and recurrent urinary tract infections (UTI). At the age of seventeen, after a routine dental procedure, a mandibular osteitis occurred that required surgical curettage. This was complicated by *Staphylococcus aureus* infection with the need of parenteral antibiotic treatment for 3 months. Also, at the age of twenty-two, tonsillectomy offered no improvement of infections (Figure 1A).

The patient was diagnosed with RA at the age of 32, with main involvement of hands and feet small joints. After transient and poor response to oral corticosteroids and conventional Disease Modifying Antirheumatic Drugs (DMARDs) such as methotrexate (MTX), leflunomide and cyclosporine, she started anti-TNF*α* therapy with Etanercept, which was discontinued after 8 months for secondary inefficacy. Other biological- DMARDs were later administered, always in combination with MTX, with poor efficacy. During this time recurrent infections continued, including vaginal candidiasis and HSV1 infections (7–8 times/year). At the age of 37 she started RTX (2 cycles/year), with good rheumatological response (Figure 1B). No immunological investigations had been performed except for a pre-RTX immunoglobulin dosage which showed low IgM (41 mg/dl) with normal IgG and IgA (793 and 170 mg/dl, respectively). Due to further worsening of infections and the observation of severe hypogammaglobulinemia (IgG 383 mg/dl, IgA 50 mg/dl, IgM 12 mg/dl mg/dl), she was referred to our Immunology Unit.

### Immunological work-up and IEI diagnosis

As reported in Table 1, an extended immunological work-up confirmed the hypogammaglobulinemia (IgG 488 mg/dl, IgA 50 mg/dl, IgM 25 mg/dl) and detected low serum free light chains (kappa sFLC 6.58 mg/L, lambda sFLC 7.45 mg/L). The immunophenotype showed a moderate increase of central memory CD4+ T cells (CD3 + CD4 + CD27 + CD45RA + 60.5%), with a remarkable reduction of effector memory and EMRA CD4 T cells (CD3 + CD4 + CD27-CD45RA- 4% and CD3 + CD4 + CD27-CD45RA + 1.3%, respectively), low naïve CD8 T cells (CD3 + CD8 + CCR7 + CD45RA + 16.7%) with expansion of the effector and terminal memory compartment (CD3 + CD8 + CCR7-CD45RA- 49,8%, CD3 + CD8 + CCR7-CD45RA + 30%). Despite normal frequencies of B cells, dramatically low switched (CD27 + IgD-IgM- 0.08%) and low IgM memory B cells (CD27 + IgD + IgM + 3.5%) were identified. Based on clinical and immunological results a presumptive diagnosis of CVID was made. Specific antibody response could not be determined since a clinical worsening during an acute infection promptly required immunoglobulin replacement treatment. After the use of RTX, the patient started a treatment with Sulfasalazine and low dose oral corticosteroids in addition to regular IgG replacement therapy, with good control of infections and autoimmunity. She is now doing well with 500 mg/kg/4 weeks of intravenous immunoglobulin (IVIG) replacement therapy (obtaining an IgG level of approximately 10 g/L) and azithromycin prophylaxis.

**Table 1:** Immunological data.

	Pt	Range
Total Lymphocytes (cell/mcl)	2,765	1,600–2,400
CD3+ (%)	78.7	61–84
CD4+ (%)	62.9	32–60
CD8+ (%)	14.2	13–40
CD19+ (%)	11.7	10–31
CD16+ (%)	9.6	3–27
Naïve CD4 (CD3 + CD4 + CD27 + CD45RA+) (%)	34.3	31–57
Central Memory CD4 (CD3 + CD4 + CD27 + CD45RA+) (%)	60.5	10–27
Effector Memory CD4 (CD3 + CD4 + CD27-CD45RA-) (%)	4	12–44
EMRA CD4 (CD3 + CD4 + CD27-CD45RA+) (%)	1.3	4–12
Naïve CD8 (CD3 + CD8 + CCR7 + CD45RA+) (%)	16.7	18–61
Central Memory CD8 (CD3 + CD4+ CCR7 + CD45RA+) (%)	3.6	3–12
Effector Memory CD8 (CD3 + CD4+ CCR7-CD45RA-) (%)	49.8	25–58
EMRA CD8 (CD3 + CD4+ CCR7-CD45RA+) (%)	30	5–20
CD19 + CD27 + IgD + IgM + (%)	3.57	4–12
CD19 + CD27 + IgD−IgM− (%)	0.08	4–16
CD21low (%)	0.4	0–6
Transitional (%)	13.7	5–15
IgG (mg/dl)	488	604–1,909
IgA (mg/dl)	50	61–301
IgM (mg/dl)	25	59–297
Kappa sFLC (mg/L)	6.58	6.7–22.4
Lambda sFLC (mg/L)	7.45	8.3–27

### Genetic characterization and functional tests

Targeting NGS analysis including 62 genes (Supplementary Table S1) causing primary antibody defects has been performed. We identified a novel heterozygous variant of uncertain significance (VUS) in *IKBKB* gene (c.1465A > G; *p*.Ser489Gly) leading to the substitution of the conserved serine in position 489 with glycine (SCV002758753 - https://submit.ncbi.nlm.nih.gov/clinvar/). This variant was not found in gnomAD exomes and genomes.

**Figure 2:**
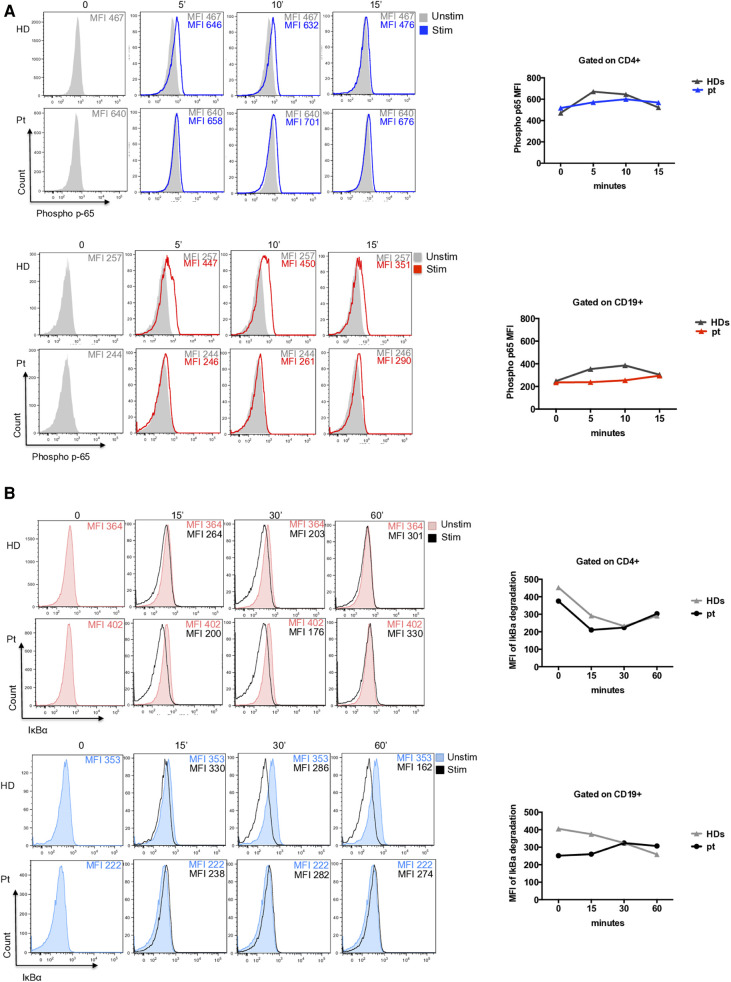
(**A**) Representative histograms of phospho-p65 expression in CD4+ T cells (blue histograms) and CD19+ B cells (red histograms) from patient and unrelated control. Graphs show the mean of two independent experiments. (**B**) Representative histograms of I*κ*B*α* degradation (a reduction of IkBa expression is showed due to degradation) in CD4+ T cells (red filled) and CD19+ B cells (blue filled) from patient and an unrelated control. Graphs show the mean of two independent experiments.

To evaluate its pathogenic role, we investigated the NF-kB pathway. The p65 phosphorylation was investigated in CD4+ T cells and CD19+ B cells at different time points after PMA/Ionomycin stimulation. We observed a delayed and reduced capacity to phosphorylate p65 overtime in CD4+ T cells (Figure 2A) and an almost absent response in CD19+ B cells (Figure 2A). Further, I*κ*B*α* degradation was normally regulated in CD4+ T cells and impaired in CD19+ B cells where protein degradation was particularly reduced (Figure 2B).

## Discussion

Here we present the case of a 47-year-old female patient who suffered with recurrent infections since early infancy, who received a diagnosis of Rheumatoid Arthritis at age 32 but refractory to several biological DMARDs before she was treated with Rituximab. In the setting of a worsening recurrence of infections, hypogammaglobulinemia was observed, and an extensive immunological work-up allowed a clinical diagnosis of Common Variable Immunodeficiency (CVID) and genetic analysis identified a novel heterozygous variant in *IKBKB* gene.

Infectious recurrence, mainly involving the respiratory tract, represent an extremely common feature during pediatric age (11) with approximately 10%–20% of children <10 years of age suffering from recurrent respiratory tract infections, requiring antibiotic treatment and, in some cases, hospitalization (12). Many children with IEI conditions may remain undiagnosed or misdiagnosed for a longtime since infections might be underestimated and each infection treated as an isolated episode while the underlying cause and appropriate investigations are missed. IEI may present at any age and with a variable clinical presentation. Approximately 25%–30% of patients with IEI may suffer with manifestations of immune dysregulation, isolated or with coexisting infections (6). Autoimmune cytopenias, rheumatologic diseases and inflammatory bowel disease as more common. In some cases, the evolution of additional medical problems complicate matters and unveiling a PIRD condition may be a daunting task. PIRD patients require a multispecialistic approach but, since the autoimmune manifestation might appear as predominant (6), these patients are often at first evaluated by non-IEI addicted specialists who might tend to manage the immune dysregulation with no vigilance on a potential underlying PIRD condition. Therefore, it's not unusual that IEI diagnosis is delayed. Also, the infectious recurrence as a potential warning sign for an IEI, may be misinterpreted as a common complication of the extended use of a wide range of biologicals/immunosuppressant treatments. Additionally, prolonged B cell deficiency have been documented in 30%–56% of patients receiving B-cell targeting agents such as RTX for the treatment of B cell lymphoproliferative diseases and autoimmune disorders (8, 13–19). Although the precise role of RTX is not easily discernible due to the heterogeneity of previous treatments, a significant increase in the percentage of patients developing isolated or combined immunoglobulin deficiency over RTX treatment has been observed for all three isotypes. Some of these patients present symptomatic hypogammaglobulinemia and require immunoglobulin replacement to prevent infectious complications (20). In our patient, at pre-RTX use IgM deficiency was detected while some years post-RTX a severe hypogammaglobulinemia occurred. In a previous study we have reported that persistent hypogammaglobulinemia after RTX may occur in a subset of children with autoimmune cytopenia, but this should not always be interpreted as iatrogenic secondary hypogammaglobulinemia since it may unveil an IEI disorder (13). Several studies have evaluated the presence of post-RTX hypogammaglobulinemia in patients with autoimmune disorders, including rheumatologic disease (14, 21–23). An imbalance between naïve and memory B cells with low switched memory B cells may mimic a CVID condition and establishing the pathogenetic role of B cell perturbation beyond a pure iatrogenic effect may be challenging, however this is not only necessary but dutiful (23).

Rheumatologic diseases are more frequently observed in patients with IEI, especially in female adults with CVID (24). However, it is not unusual to make a diagnosis of rheumatologic disease before IEI diagnosis. In our patient, either during infancy and in adulthood, a comprehensive care of the patient missed evaluation and eventually reevaluation of the patient's immune system over time for a proper and timely management.

Among PIRD-related genetic variants found in cohorts of rheumatologic patients with hypogammaglobulinemia, both monoallelic variants in NF-kB subunits deficiency and post-translational modification of NF-kB pathway proteins have been detected. These could be responsible for either immunodeficiency, autoimmune diseases (RA, SLE and inflammatory bowel diseases), or both. *IKBKB* gene is implicated in NF-*κ*B transcription signaling and particularly in the activation of the canonical NF-*κ*B pathway, which is relevant for lymphocyte activation, homeostasis and control of self-tolerance. Upon receptor ligation, signals induce activation of the IKK complex, which includes the kinases IKKalpha, IKKbeta and NEMO that are encoded by *IKBKA*, *IKBKB* and *IKBKG* genes, respectively. Then, phosphorylation of the inhibitory protein I*κ*B*α* allows release of NF-*κ*B molecules p65 (RelA), c-Rel, and p50 to the nucleus that act as transcription factors with induction of a multitude of pro-inflammatory cytokines (i.e., IL-1, IL-2, IL6, IL8, IL12 and TNF*α*), chemokines (i.e., CXCL1 and CXCL10) and other inflammatory mediators (i.e., adhesion molecules as ICAM-1, VCAM-1, ECAM-1, anti-apoptotic factors as Fas, BCL-2, Caspase, BFL-1 and cell cycle regulator as PAI2 and Cyclin) (25, 26). Thus, it is not surprising that IKK/NFKB aberrations can cause a variety of immune-related disorders.

In our patient, we identified a novel heterozygous variant of uncertain significance (VUS) in *IKBKB* gene (c.1465A > G; *p*.Ser489Gly), in a strong conserved serine in position 489. Functional analysis revealed a strongly reduced capacity to phosphorylate the p65 overtime in CD4+ T cells and an almost absent response to stimulation in CD19+ B cells. Further, I*κ*B*α* degradation was normally regulated in CD4+ T cells and impaired in CD19+ B cells. Whether this patient's functional deficiency is due to the underlying genetic defect or, alternatively, directly induced by RTX and other immunosuppressive treatments is a matter of concern. The use of immunosuppressive drugs as CCS and RTX overtime, could affect NF-kB signaling and alter T and B cell homeostasis. Only *in vitro* experiments using transducing vectors might confirm the pathogenicity of this variant.

In this scenario, it goes without saying that increasing awareness of pediatricians and other specialists who take care of these patients is warranted for an early immunology referral to prompt diagnosis and timely treatment with significant prognostic and socio-economic implications for the patient, family and community. The wider use of new laboratory-based genetic technologies and functional immune studies are leading to a better understanding of IEI/PIRDs pathophysiology. In IEI-addicted immunology centers, molecular and functional characterization of patients are started simultaneously with PIRD suspicion and with the initiation of first-line treatment for immune dysregulation. Molecular characterization of each distinct PIRD patient may expedite the therapeutic approach, by the use of immune-modifying biologicals directly targeting the altered immune pathway. This is crucial to optimize treatment efficacy and minimize possible adverse events (21). Conversely, in non-IEI addicted setting, molecular and functional characterization are explored after first and second lines of treatment (27). Thus, patients receiving RTX and/or other immunosuppressants should not exclusively regarded as affected with a iatrogenic immunodeficiency since a higher degree of suspicion for a previously undiagnosed IEI is warranted when atypical clinical and immunological features occur. In these cases, a prompt immunological referral and work-up, i.e., extensive B cell immunophenotype, Ig isotypes, vaccine responses, free serum light chains as well as evaluation and function of candidate genes by targeted NGS or whole exome/genome sequencing is recommended (10, 20). Growing knowledge on the molecular mechanisms sustaining immune dysregulation will be extremely beneficial for applying precision medicine and for continual translational progress in the field (28).

## Conclusions

In conclusion, this case highlights the importance of an early suspicion for IEI disorders since childhood and of clinical and immunological reevaluations over time for a correct interpretation of more complicated courses. Collaborations among pediatricians, expert clinicians, geneticists, are required to expand the spectrum of diagnosed IEI disorders and benefit those patients who present manifestations spanning multiple disease domains. Targeted or semi-targeted treatment options might control or balance altered immune signaling pathways, however further studies are needed to highlight the off-target effects of biologics.

## Data Availability

The datasets presented in this study can be found in online repositories. The names of the repository/repositories and accession number(s) can be found below: https://www.ncbi.nlm.nih.gov/ - SCV002758753.
